# Kanji and Kana agraphia in mild cognitive impairment and dementia: A trans-cultural comparison of elderly Japanese subjects living in Japan and Brazil

**DOI:** 10.1590/S1980-57642010DN40400008

**Published:** 2010

**Authors:** Kyoko Akanuma, Kenichi Meguro, Mitsue Meguro, Rosa Yuka Sato Chubaci, Paulo Caramelli, Ricardo Nitrini

**Affiliations:** 1 Department of Geriatric Behavioral Neurology, Tohoku University Graduate School of Medicine, Sendai, Japan.; 2 Department of Nursing, University of São Paulo School of Medicine, São Paulo SP, Brazil.; 3 Behavioral and Cognitive Neurology Unit, Department of Internal Medicine, Faculty of Medicine, Federal University of Minas Gerais, Minas Gerais BH, Brazil.; 4 Department of Neurology, University of São Paulo School of Medicine, São Paulo SP, Brazil.

**Keywords:** agraphia, mild cognitive impairment, dementia, Kanji, Kana

## Abstract

This study verifies the environmental effects on agraphia in mild cognitive impairment and dementia. We compared elderly Japanese subjects living in Japan and Brazil.

**Methods:**

We retrospectively analyzed the database of the Prevalence Study 1998 in Tajiri (n=497, Miyagi, Japan) and the Prevalence Study 1997 of elderly Japanese immigrants living in Brazil (n=166, migrated from Japan and living in the São Paulo Metropolitan Area). In three Clinical Dementia Rating (CDR) groups, i.e., CDR 0 (healthy), CDR 0.5 (questionable dementia), and CDR 1+ (dementia) , the Mini-Mental State Examination (MMSE) item of spontaneous writing and the Cognitive Abilities Screening Instrument (CASI) domain of dictation were analyzed with regard to the number of Kanji and Kana characters. Formal errors in characters and pragmatic errors were also analyzed.

**Results:**

The immigrants in Brazil wrote similar numbers of Kanji or Kana characters compared to the residents of Japan. In spontaneous writing, the formal Kanji errors were greater in the CDR 1+ group of immigrants. In writing from dictation, all the immigrant CDR groups made more formal errors in Kana than the Japan residents. No significant differences in pragmatic errors were detected between the two groups.

**Conclusions:**

Subjects living in Japan use Kanji frequently, and thus the form of written characters was simplified, which might be assessed as mild formal errors. In immigrants, the deterioration in Kanji and Kana writing was partly due to decreased daily usage of the characters. Lower levels of education of immigrants might also be related to the number of Kanji errors.

## Abstract (pt)

Este estudo verifica os efeitos do meio ambiente sobre a agrafia em comprometimento cognitivo leve e demência. Nós comparamos indivíduos idosos vivendo no Japão e Brasil.

**Métodos:**

Nós, retrospectivamente, analisamos a base de dados do Estudo de Prevalência 1998 em Tajiri (n=497, Miyagi, Japão) e do Estudo de Prevalência 1997 de imigrantes idosos japoneses vivendo no Brasil (n=166, imigrados do Japão e residindo na área metropolitana da cidade de São Paulo). Em três grupos de CDR (*Clinical Dementia Rating*), isto é, CDR 0 (saudáveis), CDR 0.5 (demência questionável) e CDR1+ (demência), o item de escrita espontânea do Mini-Exame do Estado Mental (MEEM) e o domínio de ditado do *Cognitive Abilities Screening Instrument* (CASI) foram analisados em relação ao número de caracteres em Kanji e Kana. Erros formais nos caracteres e erros pragmáticos foram também analisados.

**Resultados:**

Os imigrantes no Brasil escreveram número similar de caracteres de Kanji e Kana comparados aos residentes no Japão. Na escrita espontânea, os erros formais de Kanji foram maiores no grupo de CDR1+ em imigrantes. Na escrita sob ditado, todos os grupos de CDR de imigrantes fizeram mais erros formais em Kana do que os residentes no Japão. Nenhuma diferença foi encontrada em erros pragmáticos entre os grupos.

**Conclusões:**

Sujeitos vivendo no Japão usam Kanji freqüentemente, e então, a forma de caracteres escritos foi simplificada, o que pode ser avaliado como discretos erros formais. Em imigrantes, a deterioração na escrita em Kanji e Kana foi parcialmente devida ao uso diário restrito dos caracteres. Baixos níveis educacionais dos imigrantes podem estar relacionados ao número de erros em Kanji.

Agraphia, or impairment of the ability to write, is classified into central or peripheral types in the classic cognitive model.^1^ Namely “central agraphia” means spelling errors in legible words associated with disrupted word selection. “Peripheral agraphia” consists of mechanical distortions of writing. This includes “constructive agraphia,” which involves omissions or additions of letters.^2^ Recently, the term “dysexecutive agraphia”^3^ was proposed for a form of peripheral dysgraphia in which complex aspects of writing, such as planning, narrative coherence, and maintained attention, are significantly disturbed in cases of impairment of executive functions. Frontal lobe damaged patients not only have difficulties in maintaining the effort required for writing, but also in organizing ideas when writing texts.

Language disorders are major neuropsychiatric symptoms of degenerative dementia^4^ and Alzheimer’s disease (AD).^5^ Due to semantic memory impairment, patients with AD show “surface dysgraphia”, in which the writing of dictated words with an irregular or atypical sound-spelling correspondence (e.g. *blood*) is significantly impaired relative to regular words (e.g. *bland*).^6^ This is because irregular words generally cannot be correctly written without knowing the meaning. The majority of errors are thus phonologically plausible renderings of the target words (e.g. *honor* to ONER).^7^ Luzzatti et al.^8^ have reported multiple patterns of impairment in AD. However, no studies in a community have reported on dysgraphia of mild cognitive impairment (MCI),^9^ which is considered to be the prodromal stage of AD and other dementia.^10^

There are two kinds of scripts in the Japanese writing system, i.e., Kanji (logogram) and Kana (morphogram). Generally, Kanji words are thought to correspond to irregular words, whereas Kana words are considered to correspond to regular words in Western languages. We previously reported Kanji-predominant dyslexia, or reading impairment, in advanced AD.^11^ However, dysgraphia, especially Kanji writing impairment, should be considered in relation to educational levels and the language environment. In this regard, it would be useful to examine migrants who received education at elementary schools in the mother land before emigrating to another language environment.

Brazil is the country with the largest population of Japanese immigrants. In 1997, we surveyed elderly Japanese immigrants from Miyagi Prefecture, Japan, currently living in the São Paulo Metropolitan Area, and reported on the prevalence of dementia.^12-14^ The elderly immigrants had received education at elementary schools in Japan and emigrated to Brazil with their parents at the mean age of 14. In mostly the same year, we also surveyed Japanese elderly subjects in Tajiri, Miyagi Prefecture, Japan.^15^ The two populations were examined systematically with the same neuropsychological tests. In this study we further analyzed the two sets of data, focusing on writing tasks. This is the first comparative community-based study of agraphia in elderly Japanese subjects living in Japan and in another country with a different language environment. To study the environmental effects on agraphia in mild cognitive impairment and dementia, we compared elderly Japanese subjects living in Japan and Brazil.

## Methods

### Japanese elderly subjects in Miyagi Prefecture

We retrospectively analyzed the database of Prevalence Study 1998 (n=625) in Tajiri, Miyagi Prefecture, Japan. The detailed methodology for selecting subjects was described previously.^15^ There were 412 subjects with Clinical Dementia Rating (CDR) [16] 0 (healthy), 168 with CDR 0.5 (questionable dementia), and 45 with CDR 1+ (dementia). Compared to the CDR 0 group, the CDR 0.5 group was older and had a lower educational level, and the CDR 1+ group was older.

### Japanese elderly immigrants from Miyagi Prefecture living in the São Paulo Metropolitan Area

We also analyzed the database of Japanese elderly immigrants from Miyagi Prefecture living in the São Paulo Metropolitan Area (n=166). The detailed methodology for selecting subjects was described previously.^12-14^ They included 104 CDR 0, 49 CDR 0.5, and 13 CDR 1+ subjects, with mean ages of 76.1, 78.4, and 85.5 years, respectively. Compared with the CDR 0.5 group, the CDR 1+ group was older and had a lower educational level. Demographics of the both subjects were noted in Table 1. The immigrants in Brazil do not receive systematic learning. They received Japanese education in Japan before immigration at their mean age of 14 and their language environment had been dramatically changed. In the Japanese community they can use Kanji and Kana for writing letters, but it depends on the subjects.

**Table 1:** Demographics of two study populations.

	CDR 0				CDR 0.5				CDR 1+		
	n	age	education		n	age	education		n	age	education
Tajiri	412	72.2	8.3		168	76.4*	7.5*		45	81.4*	8.0
Immigrants	104	76.1	8.8		49	78.4	6.4		13	85.5**	5.3**

* Compared to the CDR 0 group, the CDR 0.5 group was older and had a lower educational level, and the CDR 1+ group was older (p<0.05, post hoc after ANOVA).

** Compared with the CDR 0.5 group, the CDR 1+ group was older and had a lower educational level (p<0.01, post hoc after ANOVA). CDR: Clinical Dementia Rating.

### Writing tasks

#### Spontaneous writing

The spontaneous writing item of the Mini-Mental State Examination (MMSE)^17^ was used. An A4-sized sheet of white paper was presented to the subjects for them to write whatever sentence they imagined.

#### Writing from dictation

The dictation domain of the Cognitive Abilities Screening Instrument (CASI)^18^ was used. Subjects were asked to write the dictated words “Watashi wa ie ni kaeritai” which means “I would like to go home.” This sentence is written with three Kanji and five Kana characters.

### Analyses

The numbers of Kanji and Kana characters were counted and analyzed with two-way (subjects, CDR groups) ANOVA, so did formal errors of characters. We herein defined the “formal” errors as any types of incorrect patterns of Kana and Kanji characters. They included “central agraphia” which means spelling errors in legible words associated with disrupted word selection, as well as “peripheral agraphia “which consists of mechanical distortions of writing.

## Results

### Spontaneous writing task

**Figure 1:**
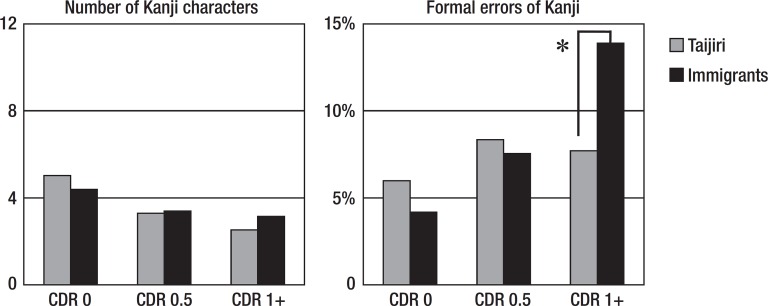
Shown are the means. *p<0.05. CDR: Clinical Dementia Rating.

Figure 1 shows the Kanji results for the spontaneous writing task. For the number of Kanji characters, a two-way ANOVA disclosed that there was no subjects (Tajiri vs immigrants) difference (F=0.917, p=0.339); however, there was a CDR group difference (F=5.669. p=0.004). For the formal errors of Kanji, there was no subjects difference (F=0.519, p=0.476); however, there was a CDR group difference (F=4.005, p=0.019). Post hoc chi-square test showed that there were more errors in the migrant CDR 1+ group (p<0.05).

**Figure 2:**
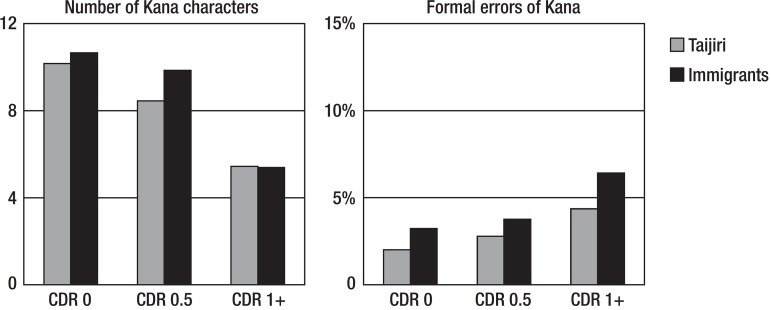
Shown are the means. CDR: Clinical Dementia Rating.

Figure 2 shows the Kana results for the spontaneous writing task. For the number of Kana characters, a two-way ANOVA disclosed that there was no subjects (Tajiri vs immigrants) difference (F=0.822, p=0.365), nor did a CDR group difference (F=1.513, p=2.222). For the formal errors of Kana, there was no subjects difference (F=0.713, p=0.399), nor did a CDR group difference (F=0.599, p=0.550). The migrants in Brazil and residents of Japan wrote a similar number of characters.

### Dictation task

**Figure 3:**
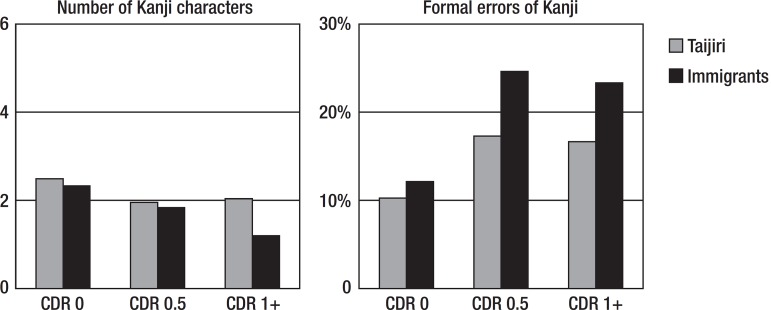
Shown are the means. CDR: Clinical Dementia Rating.

Figure 3 shows the Kanji results for the dictation task. For the number of Kanji characters, a two-way ANOVA disclosed that there was no subjects (Tajiri vs immigrants) difference (F=0.263, p=0.259); however, there was a CDR group difference (F=28.014, p<0.001). For the formal errors of Kanji, there was no subjects difference (F=0.384, p=0.536); however, there was a CDR group difference (F=5.020, p=0.007). The migrants in Brazil and residents of Japan wrote a similar number of characters, however, there tended to be more errors in the migrant CDR 0.5 and CDR 1+ groups.

**Figure 4:**
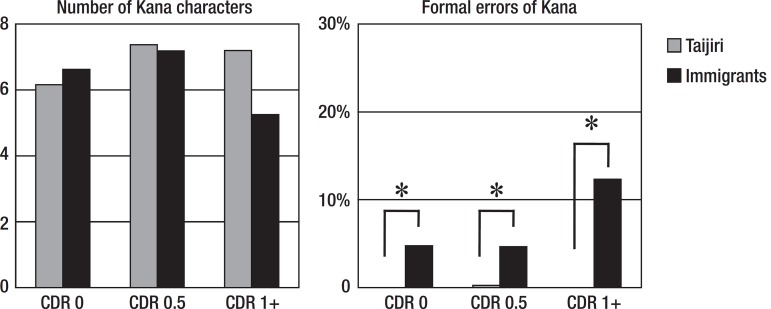
Shown are the means. *p<0.01. CDR: Clinical Dementia Rating.

Figure 4 shows the Kana results for the dictation task. For the number of Kana characters, a two-way ANOVA disclosed that there was no subjects (Tajiri vs immigrants) difference (F=3.356, p=0.068); however, there was a CDR group difference (F=14.396, p<0.001). For the formal errors of Kana, there was a subjects difference (F=21.264, p<0.001); however, there was no CDR group difference (F=2.853, p=0.059). Post hoc chi-square test showed that there were more errors in the migrant with all CDR groups (p<0.01).

“Watashi wa ie ni kaeritai” which means “I would like to go home,” of which the “wa” should be written 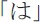 pronounced as “HA” as a particle. This was the most common error by the immigrants.

## Discussion

We should note the methodological issues first. We herein retrospectively analyzed the databases of two studies, Prevalence Study 1998 in Tajiri (Miyagi Prefecture, Japan)^15^ and Prevalence Study 1997 in the São Paulo Metropolitan Area (Japanese immigrants from Miyagi Prefecture).^12-14^ The two surveys were performed mostly in the same year, and the tasks were standardized items systematically performed in the same way. Since the two databases were from standard epidemiologic studies, we were only able to compare the MMSE item of spontaneous writing and the CASI domain of dictation. More sophisticated neuropsychological tasks would provide a better basis for comparison. Despite these limitations, we were able to provide some useful findings regarding the writing of Kanji and Kana characters in different language environments.

According to the classic cognitive model of Kanji and Kana, spontaneous writing and writing from dictation are both initiated in Wernicke’s area, which then gives rise to two separate pathways to motor areas. Kanji writing depends on the pathway that passes to the posterior inferior temporal gyrus, and then through the occipital lobe and angular gyrus. This pathway is presumed to be involved in phoneme-semantic matching (Wernicke’s area and left temporal lobe), selection of Kanji graphemes according to the meaning (left posterior inferior temporal lobe),^19^ retrieval of physical forms of the target letter (occipital lobe), and eliciting corresponding motor engrams. Kana writing may be elicited by activation in Wernicke’s area that passes directly to the angular gyrus and then to the anterior speech-motor areas.^20,21^

Usually Japanese people learn 47 Kana characters at home and learn Kanji at school. More than 2,000 Kanji are taught at elementary school. Kanji are more visually complex than Kana. The angular gyrus is thought to be associated with the recall of both Kanji and Kana characters. Our previous study indicated that the posterior inferior temporal cortex is also involved in the recall of Kanji, since this area is involved in visual information processing.

The “formal” errors in this study included “central agraphia” which means spelling errors in legible words associated with disrupted word selection, as well as “peripheral agraphia” which consists of mechanical distortions of writing. The “central agraphia” patterns were noted in immigrants probably due to their problems of Japanese Kanji and Kana words selection. The “peripheral agraphia” patterns were found in Japanese subjects, probably due to frequent usage of their own writings, seemingly assessed as the “formal errors.”

This study showed that elderly people living in Tajiri, Japan, were able to write Kana almost perfectly, with a slight deterioration in Kanji writing. They used Kanji frequently, and thus the form of written characters was slightly simplified, which might be assessed as a mild formal error. For elderly migrants from Japan living in Brazil, the deterioration of Kanji and Kana writing was partly due to less frequent daily usage of the characters. Lower levels of school education might also affect their writing, especially of Kanji characters. Also, the environment of Portuguese language and writing, and Roma-ji writing (alphabetical writing of Japanese) might also distort the writing of Kana characters.
